# Thermal conductivity of graphene nanoribbons under shear deformation: A molecular dynamics simulation

**DOI:** 10.1038/srep41398

**Published:** 2017-01-25

**Authors:** Chao Zhang, Xiao-Li Hao, Cui-Xia Wang, Ning Wei, Timon Rabczuk

**Affiliations:** 1 Division of Computational Mechanics, Ton Duc Thang University, Ho Chi Minh City, Vietnam; 2 Faculty of Civil Engineering, Ton Duc Thang University, Ho Chi Minh City, Vietnam; 3 College of Water Resources and Architectural Engineering, Northwest A&F University, 712100 Yangling, P.R. China; 4 Institute of Structural Mechanics, Bauhaus-University Weimar, 99423 Weimar, Germany

## Abstract

Tensile strain and compress strain can greatly affect the thermal conductivity of graphene nanoribbons (GNRs). However, the effect of GNRs under shear strain, which is also one of the main strain effect, has not been studied systematically yet. In this work, we employ reverse nonequilibrium molecular dynamics (RNEMD) to the systematical study of the thermal conductivity of GNRs (with model size of 4 nm × 15 nm) under the shear strain. Our studies show that the thermal conductivity of GNRs is not sensitive to the shear strain, and the thermal conductivity decreases only 12–16% before the pristine structure is broken. Furthermore, the phonon frequency and the change of the micro-structure of GNRs, such as band angel and bond length, are analyzed to explore the tendency of thermal conductivity. The results show that the main influence of shear strain is on the in-plane phonon density of states (PDOS), whose G band (higher frequency peaks) moved to the low frequency, thus the thermal conductivity is decreased. The unique thermal properties of GNRs under shear strains suggest their great potentials for graphene nanodevices and great potentials in the thermal managements and thermoelectric applications.

Graphene, a single sheet 2D material of carbon atoms, has recently attracted significant attention due to its extraordinary mechanical and electrical properties. The attractive properties of graphene are being explored for series applications including nanomechanical, nanoelectronics,etc. It has the especially excellent applicable potentials since it was experimentally accessible in 2004^1^,^2^,^3^,^4^,^5^,^6^. Graphene is regarded as an ideal heat transfer material as it is endowed with extremely high in-plane thermal conductivity^7^. The thermal conductivity of graphene can be adapted according to different requirement of different devices, such as on one hand the better radiating properties for the devices when the thermal conductivity is increased and on the other hand the more effective thermalelectric properties of the devices when the thermal conductivity is decreased. However, the properties of graphene can be influenced by defects and tailored geometry shapes (size and asymmetry), strain etc. It should be pointed out that the control of graphene with defects and doping is irreversible and unrecoverable due to that its pristine structure will break, in contrast the properties of graphene under strain can be recycled. The mechanical properties of graphene nanoribbons (GNRs) under tension and shear have been explored by experiments and atomistic simulation methods^8^,^9^,^10^,^11^,^12^,^13^,^14^. The study of Jiang *et al*. observed that the Young’s modulus is closely linked with the temperature in the certain region^15^. The Young’s modulus has been shown depending on the concentration of defects to a certain extent, and the thermal conductivity is explored to be much more sensitive than the mechanical modulus to the defects^16^,^17^. Jiang *et al*. revealed that the isotopic doping of carbon can reduce thermal conductivity of GNR remarkably by localizing phonon modes^18^. Chien *et al*. found that the thermal conductivity strongly depends on the degree of functionalization under chemisorption, which can be decreased about 80% maximum^19^. The results T.Y. Ng *et al*. indicated that the presence of the dispersed Stone–Thrower–Wales defects can decrease thermal conductivity by more than 50%^20^. Moreover, the thermal conductivity of tailored graphene shows different tendency with the mesh pores in different shapes, size, density and arrangement^21^,^22^, etc. Recently, a new structure of graphene shows the extreme reduction of the thermal conductivity by tailoring sizes in graphene nanoribbon kirigami, whose thermal conductivity is about two orders of magnitude lower than that of the corresponding GNR^23^. Furthermore, the thermal conductivity of graphene under uniaxial tensile has been verified to appear a decrease of 45% and 60% in zigzag and armchair direction^24^, respectively. Therefore, it is critical to clear the effect of strain on the thermal conductivity of such structural materials.

Strain effects on thermal conductivity of the low dimensional (two dimensional and one dimensional) materials have been studied for last at least two decades^25^,^26^,^27^. It’s reported that the thermal conductivity of the low dimensional nanostructures as the silicon and diamond nanowires and thin films is decreased continuously from compressive strain to tensile strain^27^. The thermal conductivity of GNRs is also found to appear a tendency of decreasing under tensile strain by classical molecular dynamics (MD) simulations^24^,^28^. Hence, the strain effects of graphene can play a key role in the continuous tunability and applicability of its thermal conductivity property at nanoscale, and the dissipation of thermal conductivity is a obstacle for the applications of thermal management, such as in the complementary metal-oxide-semiconductor technology and chemical vapor deposition technique. Up to now, the thermal conductivity of graphene under shear deformation has not been investigated yet. From a practical point of view, good thermal managements of GNRs have significantly potential applications of future GNR-based thermal nanodevices, which can greatly improve performances of the nanosized devices due to heat dissipations. Meanwhile, graphene is a thin membrane structure, it is also important to understand the wrinkling behavior under shear deformation. The wrinkling behavior of aluminized Kapton membranes and graphene has been verified to show an essential capability to keep its one-atom layer nanostructures stable^29^. Furthermore, graphene will form corrugation under small shear strain, and failure with bonds break when shear deformation is large enough.

In this work, we have performed computer simulations based on reverse nonequilibrium molecular dynamics (RNEMD)^30^ to investigate shear strain effects on thermal conductivity of graphene.

## Methods

The open source Large-scale Atomic/Molecular Massively Parallel Simulator (LAMMPS)^31^ is employed in our simulation. The adaptive intermolecular reactive empirical bond-order (AIREBO) potential function is employed to calculate interaction between carbon atoms in graphene, which includes both covalent bonding (stretching, bending, torsion) expressed in terms of bond orders, as well as van der Waals interactions between atoms at a larger distance:


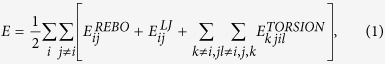


where the 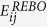 term has the same functional form as the hydrocarbon REBO potential^32^. The coefficients for 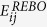 are the same as in Brenner’s potential. The 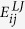 term considers the long-range interactions (2 < *r* < *cut off*) using a form similar to the standard 12–6 Lennard Jones potential, and the 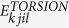 term is an explicit 4-body potential that describes various dihedral angle preferences in configurations of carbon atoms.

**Figure 1:**
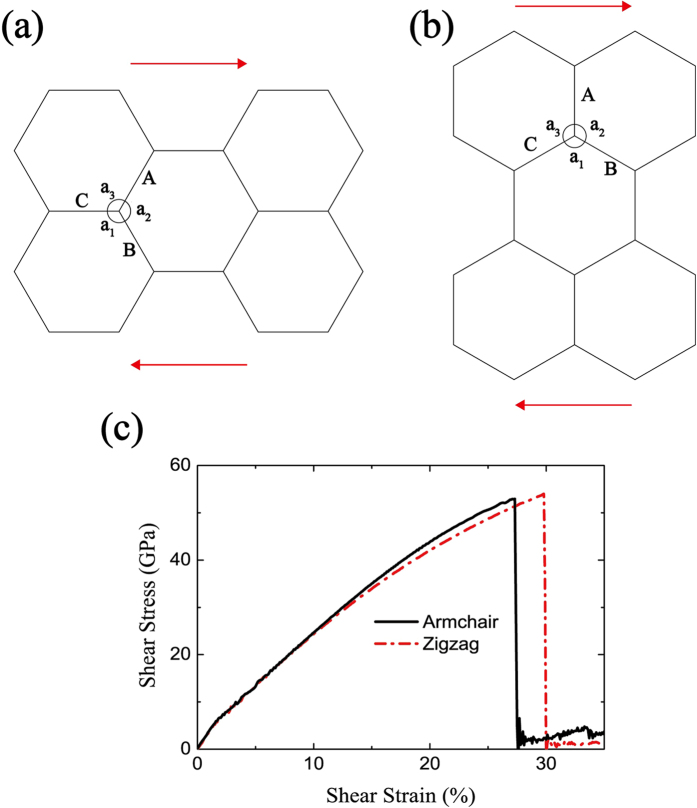
(**a**,**b**) Atomic structure of graphene along armchair and zigzag direction under shear strain, where a_1_, a_2_ and a_3_ is the bond angle, and A, B and C is the bond length, respectively. (**c**) The stress-strain curve of graphene under shear strain.

The thermal of 300 K is maintained as the background temperature by a Nos*é*-Hoover thermostat^33^,^34^. The system reaches equilibrium state in 200 picosecond (ps), and then a 0.5 femtosecond (fs) is imposed as the time step with a heat flux. The shear strains are applied according to Fig. 1(a,b). The different boundary conditions may result in different thermal conductivity. In this work, periodic boundary condition is used in all three directions..

To compute the thermal conductivity properties of sheared graphene, the size of the model is initially established in 4 nm × 15 nm. The constant pressure and constant temperature (NPT) simulation is used until the energy of the system is fully minimized. The deformation-control method^13^ is employed by applying the shear strain rate of 0.001 ps^−1^ to the bulk graphene in both zigzag graphene nanoribbons (ZGNR) and armchair graphene nanoribbons (AGNR) cases, separately. The constant volume and constant temperature (NVT) simulation is used in every 1000 time steps, which followed applied a strain increment. The time step, 0.1 fs, is applied by velocity-Verlet time stepping scheme. The shear modulus, *G*, can be gotten by the following calculation:


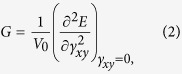


where *V*_0_ is the volume of graphene, *E* is the strain energy, and *γ*_*xy*_ is the shear strain. The shear strain expresses as *γ*_*xy*_ = Δ*x*/*L*, in which *x* and *L* are the transverse displacement and the initial length of graphene, respectively. The linear portion of the shear stress and strain curve is used,


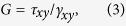


to verify the calculation of shear modulus. The *τ*_*xy*_ is the shear stress at 0.005 strain. The result here shows that the linear portion in Eq. (3) is consistent with the result obtained based on the strain energy method described as Eq. (2).

**Figure 2:**
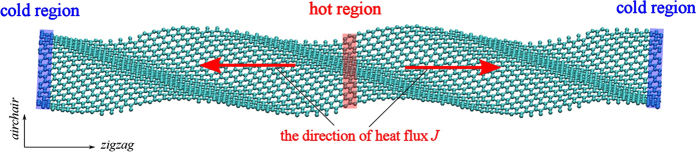
Typical example of temperature distribution which is induced by the heat flux *J*. The initial setup of the RNEMD simulation of thermal conductivity. The temperature gradient is set as a function of atomic position along the nanoribbon’s axis,Y. The thermal conductivity of GNR is then computed accordingly based on the Muller-Plathe approach^30^.

Here, we compute the thermal conductivity by using RNEMD simulation. The main idea of this method is that the model is imposed a heat flux *J* as Fig. 2 to form a temperature gradient. The advantage is that the convergence of the temperature gradient can be achieved quickly in a nonequilibrium steady system with a known quantity of heat flux. The heat flux *J* transfers energy continuously from the “hot” slab, located in the middle of the structure, to the “cold” slab, located at the beginning and end of the structure. The detail of the setup description is shown in Fig. 2. The heat flux *J* is from the heat bath to the system, and it is given by


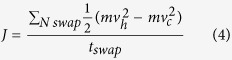


where *t*_*swap*_ and *N*_*swap*_ are the entire swap time and number of swaps, respectively; *m* is atomic mass; *v*_*h*_ and *v*_*c*_ are the velocities of the hottest and coldest atoms, respectively. The heat flux *J* is released through exchanging the momentum between the ‘hottest’ atom 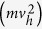 and the ‘coldest’ atom 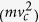. In our simulation, the structure is divided into 50 slabs along the heat transfer direction. The hot region is assigned in the 26*th* slab, and the cold region is assigned to the first slab. The two sets of atoms, hottest atoms and coldest atoms, are paired up and their velocities are exchanged. The exchange conserves both energy and momentum of the system and converges quickly in tens of picoseconds. The temperature gradient in the system is induces subsequently. The whole exchanging process is performed every 25 fs under a constant volume and constant energy (NVE) ensemble to keep the system energy conserved. The steady state can be achieved after after 200 ps of the exchanging process under NVE ensemble.

The temperature *T*_*i*_(*slab*) of of the *i*th slab is calculated based on the momentum (velocities) of all the carbon atoms in the slab as:


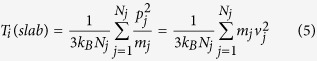


where *k*_*B*_ is Boltzmann’s constant, *N*_*j*_ is the number of the particles in the *i*th slab, *p*_*j*_ is the momentum of atom *j, m*_*j*_ and *v*_*j*_ is the mass and velocity of the *j*th carbon atom, respectively. The temperature profile is obtained by time-averaged with averaging in a time interval of 100 ps. The time-averaged temperature profile, as a function of atomic position along the nanoribbon’s axis, is used to compute the temperature gradient 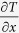. Then, the thermal conductivity of the system is calculated by using Fourier law:


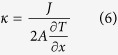


where *A* is the cross-sectional area perpendicular to the direction of heat transfer, defined by the width of GNR times the thickness of the GNR. The thickness of graphene is the carbon-carbon bond length, and the value is assumed to be 0.142 nm^28^,^35^ here. Here, the ensemble average is substituted with time averaging. In this MD simulation, the time step is 0.5 fs and the velocities are recorded every two steps. The averaging is performed over 10^5^ steps after the system reaches equilibrium for 200 ps.

## Results and Discussions

### The shear strain effect on the mechanical structure of graphene

#### Shear stress-strain curve

We study the mechanical properties of graphene along the zigzag and armchair direction under the shear strain as shown in Fig. 1. The strain at fracture along the zigzag and armchair direction is 30% and 27%, and the broken shear stress is 53.8 GPa and 53.1 GPa, respectively. The difference of anisotropic property under shear strain is less than that under uniaxial tensile. Under uniaxial tensile, the broken strain in zigzag direction of graphene is 150%^36^ of the strain in armchair direction, and broken stress in zigzag direction of graphene is 167%^36^ of the broken stress in armchair direction, respectively.

#### The change of the corrugation size under shear strain in macro view

**Figure 3:**
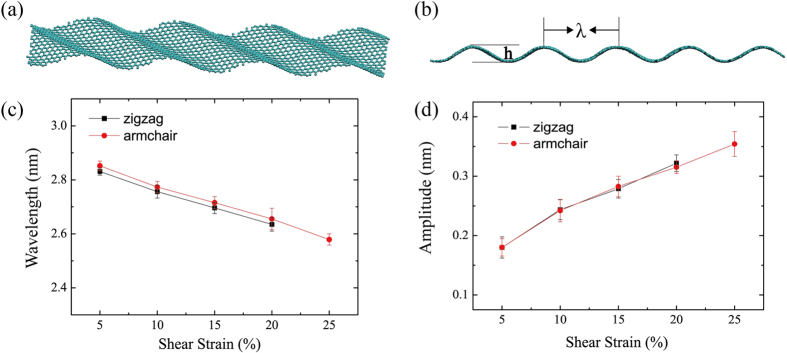
(**a**) Wrinkle structure of graphene under shear strain. (**b**) A side view of the graphene wrinkle, here the relation between the wavelength and amplitude: *λ* = *h*/2. (**c**,**d**) The relation curve of wavelength and amplitude to shear strain.

The change of amplitude and wavelength of the wrinkle graphene under different shear strain are studied. As illustrated in Fig. 3(a), shear strains cause helical wrinkles in the graphene structure. The relation of wrinkle size and shear strain is studied next. Figure 3(c,d) show the amplitude of the wave is increased as the shear strain increases, but the wavelength is decreased as the shear strain increases, probably since the difference in the bending stiffness between zigzag and armchair direction is minor. Hence, the wrinkle structure in zigzag and armchair direction under shear strain are nearly identical.

#### The micro structure change of graphene under shear strain

**Figure 4:**
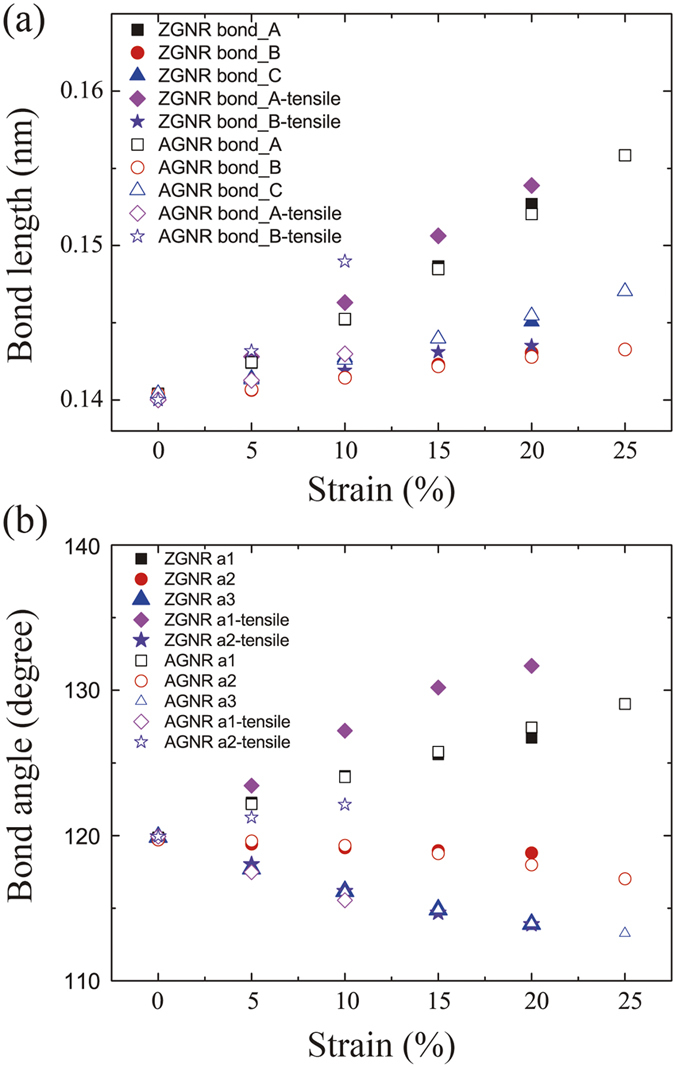
The change of the bond-angle and bond-length under shear strain. The definition of the bond-angle and bond-length is in Fig. 1(a,b). Here, (**a**) is the shear bond-length in armchair and zigzag direction. (**b**) is the shear bond-angle in armchair and zigzag direction.

The micro structure change is commonly reported based on the bond-angle and bond-length see Fig. 1. Distinct from uniaxial tensile, all the bond-angles and bond-lengths are changed independently with the shear strain, due to the broken axisymmetry. The bond-angle and bond-length due to shear strain relation are shown in Fig. 4.

The carbon-carbon bonds are elongated and angles are changed under shear stress. In order to analyze the results quantitatively, we calculated the carbon-carbon bond lengths and angles with every 0.01 strain step and the obtained values are averaged overall. We can clearly see that the bonds along the shear direction, bond type C for ZGNR and bond type A for AGNR (see Fig. 1),undergo more deformation.

Variations of bond length and bond angle of graphene are studied under shear strain in contrast with the results under uniaxial tensile. Figure 4 clearly shows the effect of shear stress to both bond-angle and bond-length are less than that of uniaxial tensile. The carbon-carbon bonds and their angles change for different values of shear stress (approximately 1.40 Å and 120° for bond length and angle, respectively without strains) is illustrated in Fig. 4. From shear stress 0.05 to 0.25 Gpa, the bond length of ZGNR and AGNR increase, in the zigzag direction, the bond type C grows faster than the bond type A, while the bond type B almost never changes; in the armchair direction, the bond type A grows faster than the bond type C. The bond type C remains nearly unaltered. The ZGNR and AGNR all have one type angle increases, but the other two types angle decrease, in the ZGNR, the angle three increase, the other angle all decrease, in the AGNR, the angle two increase, the other angle all decrease. The different changes of bond types are the reason of the different changes of angle types. These changes can also affect the thermal conductivity, and the atomic structure of graphene sheet under shear strain provides the physical insight for the mechanism of thermal transport.

### Thermal conductivity of graphene under shear strain

#### Thermal conductivity

**Figure 5:**
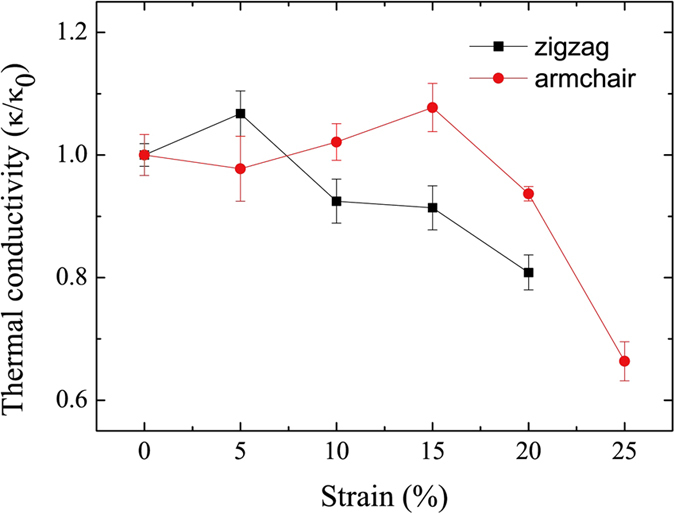
Thermal conductivity of graphene under shear strain.

Now, we study the relative thermal conductivity of graphene under shear strain. Figure 5 shows the changing process of thermal conductivity of graphene with the increase of the shear strain. With increasing shear strain coverage from 0% to 20% or 25%, the thermal conductivity increases firstly and decreases finally in both zigzag and armchair cases. At 5% coverage of the shear strain, the thermal conductivity in zigzag direction reaches the peak value, then it decreases subsequently. Different from zigzag direction, the thermal conductivity in armchair direction increases from 0% to 15% of the shear strain, and achieves its peak value at 15% of the shear strain, then it drop down rapidly. The thermal conductivity barely decreases in both zigzag and armchair direction. Compared with thermal conductivity which decrease 45% and 60%^24^ in zigzag and armchair direction under uniaxial tensile, respectively, the thermal conductivity under shear strain decreases only 12% and 16% (see Fig. 5) in zigzag and armchair direction, respectively. Thus, we conclude that the thermal conductivity of graphene is not sensitive w.r.t. shear strain. Hence, graphene nanodevices should remain stable under shear strain, but should not be used under tensile strain.

#### Phonon density of states (PDOS)

**Figure 6:**
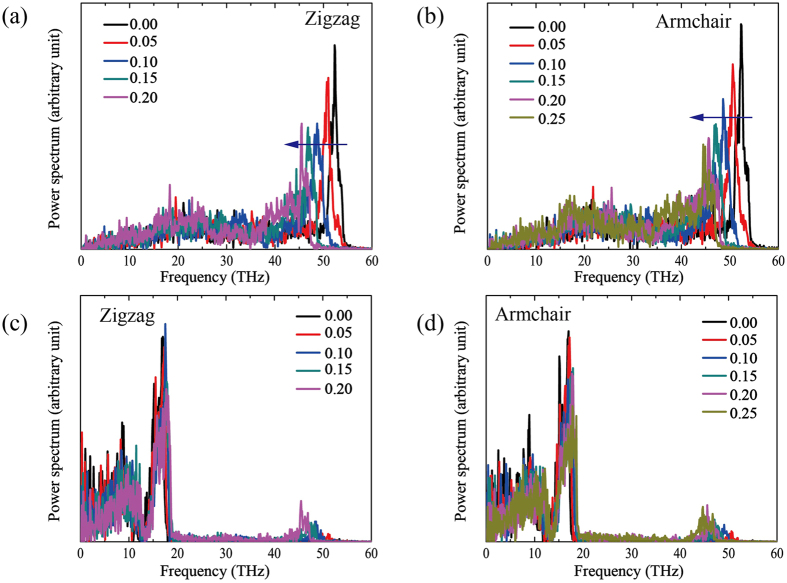
(**a**,**c**) Are the phonon density of states (PDOS) as a function of frequency with strains from 0.05 to 0.20 for ZGNR. (**b**,**d**) Are the PDOS as a function of frequency with strains from 0.05 to 0.25 for AGNR.

The strain effect on thermal transport properties is considered in this work by investigating the variation of the phonon spectra (ZGNR and AGNR cases) under different strains (see Fig. 5). The underlying mechanism of the thermal conductivity can be explained by the phonon spectra or the phonon density of states (PDOS) for the graphene under shear strain. The phonon spectrum function *G(ω*) is computed from the velocity autocorrelation function^37^ (VACF) by using Fourier transform as follows:


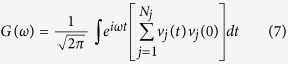


where *v*_*j*_(*t*) and *v*_*j*_(0) is the velocity of *j*th particle at time *t* and time 0, respectively, and *ω* denotes the vibrational wavenumber. The G band (higher frequency peaks) has a red shift toward the lower frequency region (see the arrow in Fig. 6). It can be seen that the G band is softened by the shear strain, and then the G band slows down the phonon group velocities. Additionally, the shear strain can produce the corrugation of graphene, which modifies the local vibrational characteristics and induces an acoustic mismatch. These appearances scatter phonons and reduce the phonons mean free path (MFP) *l*. Hence, the thermal conductivity is decreased according to the classical lattice thermal transport theory:


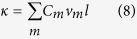


where *m* is the phonon mode value occupied at a specific temperature; *C*_*m*_, *v*_*m*_, *l* are the specific heat, group velocity and MFP of the phonon, respectively. It’s well known that the acoustic phonons are the main heat carriers in graphene near room temperature. Moreover, it was shown both theoretically and experimentally that transport properties of phonons are substantially different in graphene, which may lead to the unusual thermal conduction in graphene^38^,^39^,^40^,^41^. Additionally, the contribution of long-wavelength phonons is usually underestimated.

We further analyze the distribution of PDOS under different shear strain. It can be seen from Fig. 6, there is no clear difference of out-plane PDOS under shear strain, but the G-peak of the in-plane PDOS under shear strain shows a clear red-shift to the low frequency, which is similar to the decrease of thermal conductivity of graphene under uniaxial tensile. The tensile of the bond-length and the change of the bond-angle under shear strain causes phonon softening, followed by a decrease in the velocity of the phonon group, resulting in the decrease of the thermal conductivity. Furthermore, the change of phonon red-shift under shear strain is far less the value of uniaxial tensile, thus the decrease of the thermal conductivity is minor.

## Conclusion

In this paper, the thermal conductivity of graphene under shear strain is systematically studied by MD simulations. The results show that, in contrast to the dramatic decrease of thermal conductivity of graphene under uniaxial tensile, the thermal conductivity of graphene is not sensitive to the shear strain, and the thermal conductivity decreases only 12–16%. Studying the thermal conductivity of graphene under shear strain, we analyze the relationship of wrinkle structure of graphene sheet under shear strain. The wrinkle evolve when the shear strain is around 5–10%, but the thermal conductivity barely changes. There is evidence that the thermal conductivity of graphene does not converge even when its size reaches 9 *μ*m^42^. When the size of simulation model is smaller, considering the size effect, the wrinkle deformation could be restricted and the shape change would mainly on the atomic structure. Therefore, the thermal conductivity of graphene is more sensitive with decreasing the size of graphene. When a bigger simulation model size is employed, in contrast, the strain will express more as the wrinkle form. We found that the wrinkle has a relative small effect on the thermal conductivity of graphene in our previous research. Thus, we think that the thermal conductivity of graphene is much less sensitive to the shear strain with the size increasing. Furthermore, according to the PDOS results, we find that there is no clear difference of out-plane PDOS under shear strain, but the in-plane PDOS shows that G-peak under shear strain moves to the low frequency and shows red-shift. Since the change of phonon red-shift under shear strain is far beyond the value of uniaxial tensile, the decrease of the thermal conductivity is minor. In summary, we come to a conclusion that the thermal conductivity of graphene is not sensitive to the shear strain. Therefore, graphene nanodevices should be kept under shear strain to get relative stable thermal conductivity, and they should be avoided to be under tensile strain.

## Additional Information

**How to cite this article**: Zhang, C. *et al*. Thermal conductivity of graphene nanoribbons under shear deformation: A molecular dynamics simulation. *Sci. Rep.* **7**, 41398; doi: 10.1038/srep41398 (2017).

**Publisher's note:** Springer Nature remains neutral with regard to jurisdictional claims in published maps and institutional affiliations.

## References

[b1] Geim A. K. & Novoselov K. S. The rise of graphene. Nature materials 6, 183–191 (2007). 17330084 10.1038/nmat1849

[b2] Novoselov K. . Two-dimensional gas of massless dirac fermions in graphene. Nature 438, 197–200 (2005). 16281030 10.1038/nature04233

[b3] Zhang Y., Tan Y.-W., Stormer H. L. & Kim P. Experimental observation of the quantum hall effect and berry’s phase in graphene. Nature 438, 201–204 (2005). 16281031 10.1038/nature04235

[b4] Novoselov K. S. . Electric field effect in atomically thin carbon films. Science 306, 666–669 (2004). 15499015 10.1126/science.1102896

[b5] Stankovich S. . Graphene-based composite materials. Nature 442, 282–286 (2006). 16855586 10.1038/nature04969

[b6] Kordas K. . Chip cooling with integrated carbon nanotube microfin architectures. Applied Physics Letters 90, 123105 (2007).

[b7] Ghosh S. . Dimensional crossover of thermal transport in few-layer graphene. Nature materials 9, 555–558 (2010). 20453845 10.1038/nmat2753

[b8] Lee C., Wei X., Kysar J. W. & Hone J. Measurement of the elastic properties and intrinsic strength of monolayer graphene. Science 321, 385–388 (2008). 18635798 10.1126/science.1157996

[b9] Frank I., Tanenbaum D. M., Van der Zande A. & McEuen P. L. Mechanical properties of suspended graphene sheets. Journal of Vacuum Science & Technology B 25, 2558–2561 (2007).

[b10] Liu F., Ming P. & Li J. Ab initio calculation of ideal strength and phonon instability of graphene under tension. Physical Review B 76, 064120 (2007).

[b11] Van Lier G., Van Alsenoy C., Van Doren V. & Geerlings P. Ab initio study of the elastic properties of single-walled carbon nanotubes and graphene. Chemical Physics Letters 326, 181–185 (2000).

[b12] Zhao H., Min K. & Aluru N. Size and chirality dependent elastic properties of graphene nanoribbons under uniaxial tension. Nano letters 9, 3012–3015 (2009). 19719113 10.1021/nl901448z

[b13] Zhao H. & Aluru N. Temperature and strain-rate dependent fracture strength of graphene. Journal of Applied Physics 108, 064321 (2010).

[b14] Min K. & Aluru N. Mechanical properties of graphene under shear deformation. Applied Physics Letters 98, 013113 (2011).

[b15] Jiang J.-W., Wang J.-S. & Li B. Young’s modulus of graphene: a molecular dynamics study. Physical Review B 80, 113405 (2009).

[b16] Hao F., Fang D. & Xu Z. Mechanical and thermal transport properties of graphene with defects. Applied physics letters 99, 041901 (2011).

[b17] Zhao J. . Thermal conductivity of carbon nanocoils. Applied Physics Letters 103, 233511 (2013).

[b18] Jiang J.-W., Lan J., Wang J.-S. & Li B. Isotopic effects on the thermal conductivity of graphene nanoribbons: Localization mechanism. Journal of Applied Physics 107, 054314 (2010).

[b19] Chien S.-K., Yang Y.-T. . Influence of chemisorption on the thermal conductivity of graphene nanoribbons. Carbon 50, 421–428 (2012).

[b20] Ng T., Yeo J. & Liu Z. A molecular dynamics study of the thermal conductivity of graphene nanoribbons containing dispersed stone–thrower–wales defects. Carbon 50, 4887–4893 (2012). 10.1088/0957-4484/23/38/385702 22947664

[b21] Hu L. & Maroudas D. Thermal transport properties of graphene nanomeshes. Journal of Applied Physics 116, 184304 (2014).

[b22] Yarifard M., Davoodi J. & Rafii-Tabar H. In-plane thermal conductivity of graphene nanomesh: A molecular dynamics study. Computational Materials Science 111, 247–251 (2016).

[b23] Wei N. . Thermal conductivity of graphene kirigami: ultralow and strain robustness. Carbon 104, 203–213 (2016).

[b24] Wei N., Xu L., Wang H.-Q. & Zheng J.-C. Strain engineering of thermal conductivity in graphene sheets and nanoribbons: a demonstration of magic flexibility. Nanotechnology 22, 105705 (2011). 21289391 10.1088/0957-4484/22/10/105705

[b25] Bhowmick S. & Shenoy V. B. Effect of strain on the thermal conductivity of solids. The Journal of chemical physics 125, 164513 (2006). 17092111 10.1063/1.2361287

[b26] Picu R., Borca-Tasciuc T. & Pavel M. Strain and size effects on heat transport in nanostructures. Journal of applied physics 93, 3535–3539 (2003).

[b27] Li X., Maute K., Dunn M. L. & Yang R. Strain effects on the thermal conductivity of nanostructures. Physical Review B 81, 245318 (2010).

[b28] Guo Z., Zhang D. & Gong X.-G. Thermal conductivity of graphene nanoribbons. Applied physics letters 95, 163103 (2009).

[b29] Wang C., Mylvaganam K. & Zhang L. Wrinkling of monolayer graphene: a study by molecular dynamics and continuum plate theory. Physical Review B 80, 155445 (2009).

[b30] Müller-Plathe F. A simple nonequilibrium molecular dynamics method for calculating the thermal conductivity. The Journal of chemical physics 106, 6082–6085 (1997).

[b31] Plimpton S. Fast parallel algorithms for short-range molecular dynamics. Journal of computational physics 117, 1–19 (1995).

[b32] Brenner D. W. . A second-generation reactive empirical bond order (rebo) potential energy expression for hydrocarbons. Journal of Physics: Condensed Matter 14, 783 (2002).

[b33] Nosé S. A unified formulation of the constant temperature molecular dynamics methods. The Journal of chemical physics 81, 511–519 (1984).

[b34] Hoover W. G. Canonical dynamics: equilibrium phase-space distributions. Physical Review A 31, 1695 (1985). 10.1103/physreva.31.1695 9895674

[b35] Xu Z. & Buehler M. J. Strain controlled thermomutability of single-walled carbon nanotubes. Nanotechnology 20, 185701 (2009). 19420624 10.1088/0957-4484/20/18/185701

[b36] Wei N. . Knitted graphene-nanoribbon sheet: a mechanically robust structure. Nanoscale 4, 785–791 (2012). 22170502 10.1039/c1nr11200g

[b37] Alaghemandi M., Leroy F., Müller-Plathe F. & Böhm M. C. Thermal rectification in nanosized model systems: A molecular dynamics approach. Physical Review B 81, 125410 (2010).

[b38] Balandin A. A. Thermal properties of graphene and nanostructured carbon materials. Nature materials 10, 569–581 (2011). 21778997 10.1038/nmat3064

[b39] Nika D. L. & Balandin A. A. Two-dimensional phonon transport in graphene. Journal of Physics: Condensed Matter 24, 233203 (2012). 22562955 10.1088/0953-8984/24/23/233203

[b40] Chen S. . Thermal conductivity of isotopically modified graphene. Nature materials 11, 203–207 (2012). 22231598 10.1038/nmat3207

[b41] Nika D. L., Askerov A. S. & Balandin A. A. Anomalous size dependence of the thermal conductivity of graphene ribbons. Nano letters 12, 3238–3244 (2012). 22612247 10.1021/nl301230g

[b42] Xu X. . Length-dependent thermal conductivity in suspended single-layer graphene. Nature communications 5, 3689 (2014). 10.1038/ncomms4689 24736666

